# Reflections on the domino effect of a patient with multimorbidity: A case report and literature review

**DOI:** 10.1097/MD.0000000000042004

**Published:** 2025-05-09

**Authors:** Meizi Guo, Yu Wang, Kai Chen

**Affiliations:** a Department of General practice, Shenzhen Hospital of Southern Medical University, Shenzhen, China.

**Keywords:** comorbidity, domino effect, multimorbidity, nonocclusive mesenteric ischemia (NOMI)

## Abstract

**Rationale:**

As China enters an accelerated stage of global population aging, multimorbidity has emerged as a significant public health challenge. Current management strategies often focus primarily on index diseases, neglecting the complex interplay of multiple conditions in elderly patients.

**Patient concerns:**

An 86-year-old female with multimorbidity, including diabetes, hypertension, and osteoporosis, sustained a femoral fracture. Following “artificial bone replacement” surgery, she experienced significant loss of consciousness with a drastic decline in hemoglobin from 110 to 39 g/L.

**Diagnoses:**

The patient’s femoral fracture triggered a cascade of complications, resulting in hypoglycemic coma, gastrointestinal bleeding, and acute cerebral infarction. Utilizing a multimorbidity framework, general practitioners hypothesized and subsequently confirmed nonocclusive mesenteric ischemia (NOMI) as the cause of bleeding.

**Interventions:**

Management included timely interventions focused on addressing the underlying NOMI, specifically volume expansion and vasodilator therapy.

**Outcomes:**

Following the targeted interventions addressing both the primary conditions and complications, the patient recovered sufficiently to be discharged from the hospital.

**Lessons:**

This case underscores the importance of holistic approaches in clinical practice when managing elderly patients with multimorbidity. It demonstrates how multiple conditions can interact in a domino effect and highlights the value of considering less common diagnoses like NOMI in complex elderly patients with multiple comorbidities.

## 1. Introduction

Epidemiologic studies revealed that more than 50% of the elderly in China have 2 or more chronic health problems at the same time.^[1]^ The coexistence of multiple diseases can increase the risk of polypharmacy and disability, reduce treatment compliance, and lead to a poor quality of life,^[2,3]^ which is one of the medical problems that cannot be ignored in an aging society. However, there are currently few studies about it, and even no consensus on terminology. The terms comorbidity and multimorbidity are the most widely used in our country.^[3]^ The former normally refers to a certain index disease and its coexisting other chronic health problems, while the latter often represents 2 or more chronic health problems at the same time, which are equally important and collectively affect people. The 2 terms are similar but provide different perspectives and ideas for health management.

In clinical practice, elderly patients often present with multiple equally important acute or chronic health problems to be addressed in a single consultation. Under such circumstances, the clinical thinking of multimorbidity has more advantages, while the comorbidity perspective may lead to excessive focus on index diseases, neglecting other health problems. This article presents a case of an elderly patient with multimorbidity harmed by a domino effect resulting from hypoglycemic coma, gastrointestinal bleeding, and acute cerebral infarction. Analyzing the patient’s diagnosis and treatment process reveals that more attention was paid to single diseases while other coexisting health problems were overlooked, causing an increase in the number of medications and drug interactions, which contributed to the domino effect in this patient. The multimorbidity framework can address this oversight, helping clinicians adopt a more holistic assessment and implement more appropriate treatment strategies.

## 2. Case

Written informed consent was obtained from the patient for the publication of this case report. The patient was an 86-year-old woman who came to the emergency department of a private hospital on August 5, 2022. Eight hours prior, she suddenly became unconscious and vomited a small amount of white foam, but without symptoms of incontinence or convulsions. Upon arrival, her capillary glucose level was 1.2 mmol/L; 50% glucose was injected intravenously immediately, followed by 5% glucose for maintenance treatment. Three hours later, she regained consciousness, and a retest of her blood glucose level showed 6 mmol/L. She was then transferred to the emergency ward for observation.

The patient had a relevant clinical history of type 2 diabetes mellitus and hypertension for approximately 20 years and had been taking metformin (0.5 g bid) and glipizide sustained-release tablets (5 mg qd) to control her blood glucose for the past 5 years. Ten days prior, she had undergone surgery for a right femoral neck fracture and was on rivaroxaban (10 mg qd) for anticoagulation and diclofenac (75 mg bid) for pain management post-surgery.

**Figure 1.**
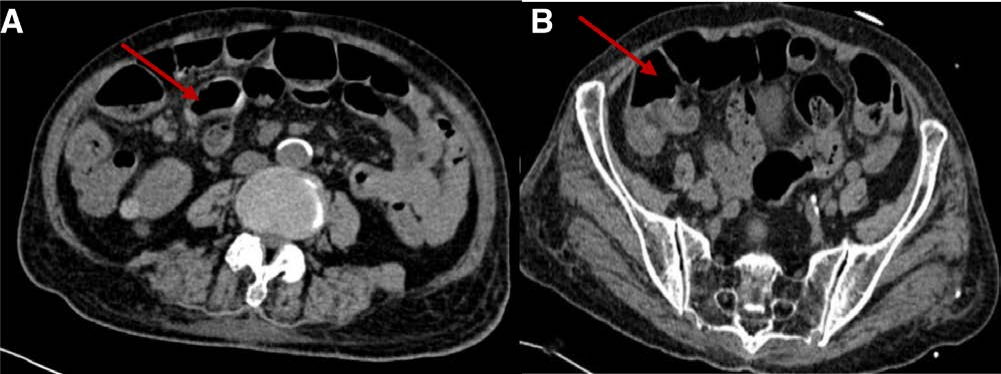
Abdominal CT scan views showing partial dilated small intestine (A) and colon (B) with effusion and multiple fluid levels (arrows). CTA = computed tomography angiogram.

On physical examination, she was alert and oriented, with mild tenderness in the right epigastric region but no signs of peritonitis. The remainder of the physical examination did not reveal any significant findings. Laboratory investigations indicated infection and anemia (Neutrophil: 93.2%, hemoglobin [Hb]: 55 g/L, C-reactive protein: 270 mg/L). A head computed tomography (CT) scan showed multiple cerebral ischemic lesions and lacunar infarction, while an abdominal CT scan revealed a partially dilated small intestine and colon with multiple fluid levels (Fig. 1).

During the observation period, the patient began a normal diet alongside 5% glucose (1000 mL iv, qd), but she experienced hypoglycemia 6 times within 24 hours, with the lowest blood glucose level recorded at 1.6 mmol/L. Additionally, she exhibited coffee-colored stool, and a fecal occult blood test was positive (+++). Immediate hemostatic therapy with snake venom hemocoagulase and aminocaproic acid sodium chloride was initiated. However, she subsequently developed abdominal pain and reported bloody stools 2 to 3 times/d, leading to a drop in her Hb level to 47 g/L, prompting her transfer to the general practice ward for further treatment.

**Figure 2.**
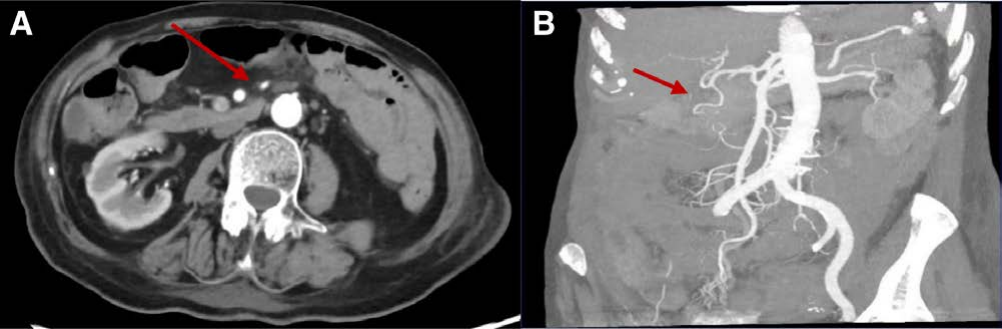
Abdominal CTA shows the superior mesenteric (A) and right renal artery artery (B) with mild atherosclerosis (arrows). CTA = computed tomography angiogram.

**Figure 3.**
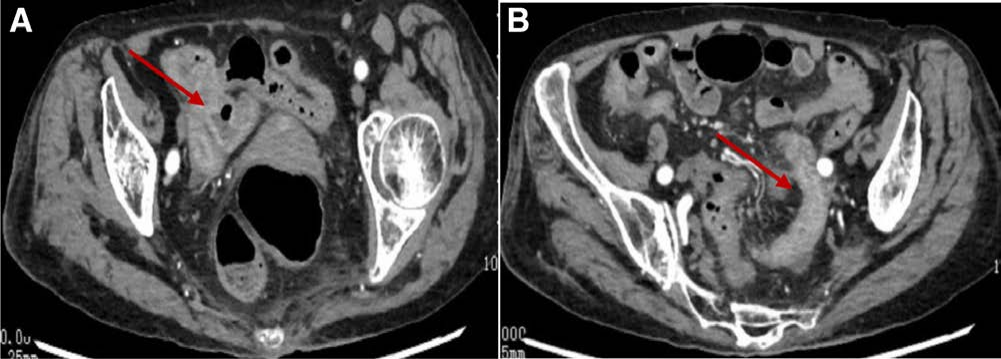
Abdominal enhanced CT shows the partial mildly dilated colon with multiple fluid levels (A) and the edematous and thickened intestinal wall of the ileum (A; arrows). CT = computed tomography.

The patient then exhibited mental changes, including gibberish, which led to an MRI examination revealing a small amount of acute cerebral infarction in the right cerebellum and left parietal lobe. Her Hb continued to decline, reaching a low of 39 g/L. To investigate the cause of the bleeding, a computed tomography angiogram (CTA) of the abdomen was performed, revealing mild atherosclerosis in the superior mesenteric and right renal arteries, but no hemorrhagic regions (Fig. 2). An abdominal CT enhancement showed a partially dilated and edematous colon with multiple fluid levels, along with a thickened ileal wall (Fig. 3). Based on the patient’s medical history, the general practitioners considered nonocclusive mesenteric ischemia (NOMI) as the likely cause of the bleeding.

**Figure 4.**
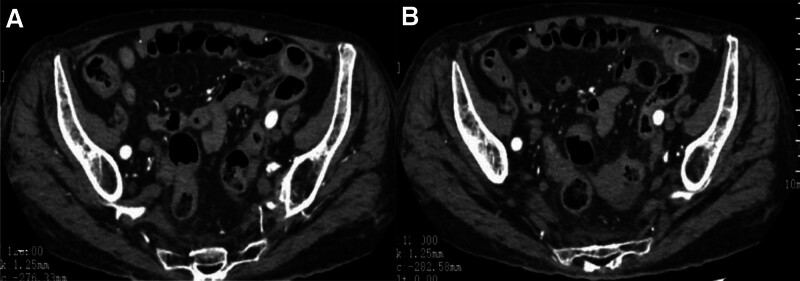
After treatment, reexamination of abdominal CT shows no obvious abnormalities (A and B). CT = computed tomography.

Instead of the current treatment, the patient received papaverine hydrochloride for vasodilation and supportive therapy to improve perfusion and oxygenation. By August 14, 2023, she reported no further episodes of bloody or dark stools. On August 18, her Hb increased to 82 g/L, and an abdominal CT enhancement showed no obvious abnormalities (Fig. 4). She was discharged from the hospital on August 22, after the fecal occult blood test returned negative for 2 consecutive tests and her blood glucose levels stabilized.

## 3. Discussion

The patient in this case primarily suffers from 2 chronic diseases: diabetes and hypertension. Additionally, she has an undiagnosed condition of osteoporosis. Following an accident, she fell while walking on flat ground, resulting in a fracture of her femoral neck, with digital imaging confirming that osteoporosis was the main “culprit.” A meta-analysis by Bigoni et al indicates that the incidence of osteoporosis among the elderly in China is 37.7%, rising to as high as 51.8% in those aged ≥80 years old.^[4]^ In older adults, a femoral neck fracture is often referred to as “the last fracture in life,” associated with nearly 50% mortality.^[5]^

The fragility fracture in this case served as the initial instigator of a domino effect of health issues. However, like many elderly individuals, this patient had never undergone screening for fracture risk or received a diagnosis of osteoporosis prior to her fracture. This highlights the necessity for increased awareness and attention to osteoporosis, as well as the importance of active health management for populations at high-risk. Regular assessments of fracture risk could help prevent fragility fractures, thereby averting the initiation of a cascade of adverse health events.

The patient in this case mainly has 2 chronic diseases: diabetes and hypertension. She also suffers from a previously undiagnosed condition: osteoporosis. In an accident, she fell while walking on flat ground, resulting in a fracture of her femoral neck, with digital imaging confirming osteoporosis as the main “culprit.” A meta-analysis by Bigoni et al indicates that the incidence of osteoporosis in the elderly in China is 37.7%, and as high as 51.8% in those aged ≥80 years.^[4]^ Femoral neck fractures are often referred to as “the last fracture in life” due to their high-mortality rate, approaching 50%.^[5]^ The fragility fracture is the instigator in our case; however, like many elderly individuals, this patient had never been screened for fracture risk or diagnosed with osteoporosis prior to the incident. Therefore, emphasizing osteoporosis management, implementing active health monitoring for high-risk populations, and regularly assessing fracture risk could prevent fragility fractures, thereby averting the initiation of the disease domino effect.

In this case, the orthopedist focused solely on the diagnosis and treatment of fractures, leading to the prescription of diclofenac for pain relief post-“artificial bone replacement” surgery. This oversight disregarded potential drug interactions. Diclofenac can compete with sulfonylureas (i.e., glipizide), which the patient had been using to control her blood glucose for an extended period. The concurrent use of these medications can significantly elevate plasma-free concentrations of glipizide, predisposing the patient to hypoglycemia and subsequent loss of consciousness. Despite timely treatment, the patient experienced 6 episodes of hypoglycemia within 24 hours, with the lowest recorded blood glucose level at 1.6 mmol/L. This may be attributed to a reduced glucose drip rate when blood glucose levels rose to 6 mmol/L. The hypoglycemia induced by medium- and long-term sulfonylureas such as glipizide often persists and is challenging to correct, necessitating continuous intravenous glucose infusion for typically more than 72 hours. Thus, the hypoglycemic event marked the 2nd domino to fall during the progression of her condition.

Following the hypoglycemic episodes, the patient began experiencing abdominal pain and bloody stools, with a precipitous drop in Hb levels. Initially, gastrointestinal bleeding was presumed to be a result of her medication regimen, including rivaroxaban and diclofenac. However, even after discontinuing these drugs and supplementing with hemostatic therapy, bleeding continued. With a comprehensive assessment rooted in multimorbidity thinking, the general practitioners speculated that the cause of bleeding was ischemic bowel disease (ICBD), a condition characterized by insufficient splanchnic circulation, preventing the intestines from receiving adequate blood and oxygen. Severe hypoglycemia can stimulate the adrenergic nervous system, exacerbating mesenteric vasospasm and diminishing blood flow to the bowel wall, ultimately leading to ICBD.^[6–8]^ The Chinese 2011 guidelines classify ICBD into acute mesenteric ischemia, chronic mesenteric ischemia, and ischemic colitis.^[9]^ Acute mesenteric ischemia may be occlusive (due to arterial embolism, thrombosis, or mesenteric venous thrombosis) or NOMI, which occurs with mesenteric arteriosclerosis without obstructed arteries or veins.^[10,11]^ Currently, CTA of the abdomen is regarded as the gold standard for diagnosing ICBD, boasting a sensitivity of 85% to 98% and specificity of 91% to 100%.^[12,13]^ In this case, CTA revealed mesenteric arteriosclerosis, while abdominal CT enhancements indicated a mildly dilated colon with multiple fluid levels and thickened ileal walls, supporting the diagnosis of NOMI.

NOMI treatment focuses on rectifying the underlying cause, enhancing bowel perfusion, and alleviating arterial spasms. The mainstays of therapy include volume expansion with fluid resuscitation, optimization of hemodynamic status, discontinuation of vasopressors, and use of anticoagulation. Consequently, rather than employing hemostatic drugs, the patient’s condition improved rapidly with volume expansion and vasodilator treatment, further confirming the diagnosis of NOMI. It is noteworthy that NOMI is more prevalent in individuals over 50 and is linked with severe hypoglycemia, diabetes, or atherosclerotic disease due to reduced arterial vascular resistance and vasoconstriction of mesenteric arteries amid circulatory failure. Thus, gastrointestinal bleeding represented the 3rd domino falling in the disease’s progression.

Atherosclerosis, high platelet aggregation, and increased blood viscosity are common among elderly diabetic patients. Upon experiencing hypoglycemia, a marked reduction in cerebral blood supply occurs, causing enhanced platelet adhesion and aggregation, which creates a vicious cycle. Furthermore, severe hypoglycemia can disrupt energy metabolism in the brain and trigger reflexive sympathetic stimulation, resulting in cerebral vasospasm and subsequent brain tissue hypoperfusion. These factors collectively contribute to the onset of cerebral infarction, marking it as the 4th domino in the progression of this patient’s condition.

The fragility fracture served as the initial trigger, followed by a domino effect of hypoglycemic coma, gastrointestinal bleeding, and acute cerebral infarction. It is essential to conduct careful and regular assessments of osteoporosis risk to avert bone fractures. Additionally, glipizide should be substituted with α-glucosidase inhibitors, dipeptidyl peptidase-4 inhibitors, sodium-glucose co-transporter-2 inhibitors, or other medications with a lower hypoglycemia risk, especially when administering diclofenac for fractures. The Chinese clinical guidelines for managing diabetes in the elderly stress the importance of maximizing benefits and establishing individualized control goals.^[14]^ Factors such as prolonged diabetes duration, impaired pancreatic β-cell function, and heightened hypoglycemia risks with insulin secretagogues necessitate more lenient blood glucose control strategies to prevent severe hypoglycemic events.^[15]^ Lastly, NOMI often lacks typical clinical presentations due to unobstructed arteries and veins, yet remains a high-mortality condition despite treatment advances, warranting greater attention.^[16]^ Initial management should encompass volume resuscitation with crystalloids to enhance perfusion and oxygenation, avoiding hemostatic drugs that could exacerbate gastrointestinal bleeding and potentially result in mortality.

Most importantly, while previous studies have highlighted the challenges posed by polypharmacy treatment in elderly patients with multiple complex diseases,^[17]^ there is a notable scarcity of research focusing on the concept of multimorbidity. It is crucial for clinicians to adopt multimorbidity thinking more frequently, particularly in considering potential cross-reactions between various medications in the elderly.^[18]^

There are potential limitations to our case report. Firstly, it is a single case study and may not be representative of all patients with multimorbidity. We have also acknowledged the challenges in generalizing the findings due to the unique nature of the patient’s clinical presentation and the complexity of her medical history. In addition, this is a retrospective analysis and there may be bias in interpreting the course of patient care.

## 4. Conclusion

Aging is increasingly pronounced in China, and the coexistence of multiple diseases poses significant threats to the physical health of elderly patients. The concept of multimorbidity can help mitigate the risks associated with polypharmacy and disability, enhance treatment compliance, and improve the quality of life. However, many clinicians have yet to fully embrace multimorbidity thinking, and the limited number of cases or studies addressing it underscores the need for increased attention in future research.

## Acknowledgments

We want to thank everyone at the department of general practice, and the patient for allowing us to share her story and publish her case report. Yu Wang designed the study. Meizi Guo wrote the original draft. Yu Wang collected raw data. Kai Chen performed statistical and bioinformatics analyses. Meizi Guo supervised the study.

## Author contributions

**Conceptualization:** Meizi Guo.

**Methodology:** Yu Wang, Kai Chen.

**Supervision:** Meizi Guo.

**Writing – original draft:** Meizi Guo, Yu Wang, Kai Chen.

**Writing – review & editing:** Meizi Guo.
